# Peri-operative diaphragm ultrasound as a new method of recognizing post-operative residual curarization

**DOI:** 10.1186/s12871-021-01506-3

**Published:** 2021-11-19

**Authors:** Jiaxin Lang, Yuchao Liu, Yuelun Zhang, Yuguang Huang, Jie Yi

**Affiliations:** 1 Department of Anesthesiology, Chinese Academy of Medical Science, Peking Union Medical College Hospital, No 1, Shuaifuyan, Dongcheng district, Beijing, 100730 China; 2 Medical Research Center, Chinese Academy of Medical Science, Peking Union Medical College Hospital, Beijing, 100730 China

**Keywords:** Diaphragm ultrasound, Diagnostic test, Neuromuscular monitor, Train-of-four, Post-operative residual Curarization

## Abstract

**Background:**

This study sought to evaluate the diagnostic accuracy of peri-operative diaphragm ultrasound in assessing post-operative residual curarization (PORC).

**Methods:**

Patients undergoing non-thoracic and non-abdominal surgery under general anaesthesia were enrolled from July 2019 to October 2019 at Peking Union Medical College Hospital. A train-of-four ratio (TOFr) lower than 0.9 was considered as the gold standard for PORC. Diaphragm ultrasound parameters included diaphragmatic excursion (DE) and diaphragm thickening fraction (DTF) during quiet breathing (QB) and deep breathing (DB). The diaphragm excursion fraction (DEF) was calculated as the DE-QB divided by the DE-DB. The diaphragm excursion difference (DED) was defined as DE-DB minus DE-QB. Receiver operating characteristic curve analysis was used to determine the cut-off values of ultrasound parameters for the prediction of PORC.

**Results:**

In total, 75 patients were included, with a PORC incidence of 54.6%. The DE-DB and DED were positively correlated with the TOFr, while the DEF was negatively correlated with the TOFr. The DE-DB cut-off value for predicting PORC was 3.88 cm, with a sensitivity of 85.4% (95% confidence interval [CI]: 70.1–93.9%), specificity of 64.7% (95% CI: 46.4–79.7%), positive likelihood ratio of 2.42 (95% CI 1.5–3.9), and negative likelihood ratio of 0.23 (95% CI: 0.1–0.5). The DED cut-off value was 1.5 cm, with a specificity of 94.2% (95% CI: 80.3–99.3%), sensitivity of 63.4% (95% CI: 46.9–77.9%), positive likelihood ratio of 10.78 (95% CI: 2.8–42.2), and negative likelihood ratio of 0.39 (95% CI: 0.3–0.6).

**Conclusions:**

Peri-operative diaphragm ultrasound may be an additional method aiding the recognition of PORC, with DED having high specificity.

## Background

Post-operative residual curarization (PORC) remains an essential clinical challenge, with an incidence ranging from 7 to 88% [1]. Residual blockade leads to an increased risk of respiratory complications, including airway obstruction, hypoxia, and reintubation, as well as to prolonged lengths of stay in the post-anaesthesia care unit (PACU) [2–4]. Neuromuscular monitoring of the train-of-four ratio (TOFr) at the adductor pollicis is considered a gold standard in reflecting sufficient recovery from the neuromuscular blockade, whereby a patient is considered to have sufficiently recovered if the TOFr is above 0.9 [5]. However, due to complicated procedures, the requirement of specific equipment, ease of interference, and inconvenience of the test, the use of a neuromuscular monitor remains clinically restricted [6], especially in China [7]. Many Chinese hospitals cannot afford to equip neuromuscular monitors in every operating room due to limited medical funding. The incidence of PORC remains quite high. Thus, it is important to investigate new ways to detect PORC when neuromuscular monitoring equipment is inaccessible.

The diaphragm is a major respiratory muscle, accounting for 60–70% of the respiratory workload. Its dysfunction involves post-operative respiratory failure, especially in the context of prolonged mechanical ventilation [8, 9]. Ultrasound is a non-invasive and visible method of assessing diaphragm morphology in both healthy volunteers [10] and intensive care unit (ICU) patients [11], representing a reproducible, feasible, and valid [12, 13] technique, according to previous research. Diaphragm ultrasound (DUS) parameters, including diaphragmatic excursion (DE) and diaphragm thickening fraction (DTF), correlate to inspiratory nasal pressure and transdiaphragmatic pressure in spontaneous respiration [14–17]. As such, DUS can be used as a substitute to predict diaphragm muscle strength, since direct measurement would be otherwise invasive and likely to incur severe complications.

The use of DUS in the evaluation of diaphragm involvement in neuromuscular disease and in the prediction of weaning mechanical ventilation in the ICU has been reported recently [18]. The peri-operative examination of diaphragm function is of great value, but is seldom performed in the operating room.

The purpose of this study was to assess the diagnostic accuracy of ultrasound parameters in recognizing residual neuromuscular blockade, using TOFr as the reference standard, in patients receiving general anaesthesia with nondepolarizing neuromuscular blockade for non-thoracic and non-abdominal surgery.

## Materials and methods

### Participants

This was a prospective observational research study approved by the Institutional Review Board (IRB) of the Peking Union Medical College Hospital (PUMCH) on May 21, 2019 (ZS-1984). Written informed consent was obtained from all subjects before pre-operative evaluation by an anaesthesiologist. This manuscript adheres to the applicable STARD [19] guidelines.

Patients scheduled for elective non-abdominal and non-thoracic surgery in the PUMCH who were administered anaesthesia by a specific anaesthesiologist were consecutively enrolled every Thursday in a selected operation room. All patients aged 18–65 years with an American Society of Anesthesiologists (ASA) physical status classification of I or II were recruited.

### Anaesthesia protocol

Anaesthesia was induced with fentanyl 2 μg/kg, midazolam 1 mg, and propofol 1–2 mg/kg, after blood pressure, electrocardiography, and pulse oxygen saturation (SpO_2_) were monitored and an intravenous cannula was established. Neuromuscular monitoring was calibrated and stabilized before rocuronium (0.6 mg/kg) administration. After intubation, inhaled anaesthetic sevoflurane combined with 50% nitrous oxide in oxygen was used to maintain a minimal alveolar concentration within the range of 0.9–1.2 during the operation. Fentanyl, remifentanil, and rocuronium were administered as necessary. The administration of neuromuscular blocking drugs was ceased approximately 30 min prior to the end of surgery. When the anaesthesiologist determined that the patient had adequately regained consciousness, myodynamia, respiratory function, and airway protection, the patient was extubated.

The TOFr within 1 minute before extubation was recorded. Post-operative DUS was performed immediately after extubation, such that the time interval between the TOFr before extubation and post-operative DUS parameters was less than 2 min. The modified observer’s assessment of alert/sedation (OAA/S) score immediately after extubation was recorded. The anaesthesiologist was blinded to the TOFr results to prevent researcher bias. After tracheal extubation, the patients immediately underwent DUS, and were transferred to the PACU. The modified Aldrete score was evaluated in the PACU, 15 min after extubation [20].

Patient demographic data, neuromuscular blocking agent dose, total opioid consumption, duration of surgery, and reintubation events were recorded. The patients were followed up for 1 month for post-operative pulmonary complications, including upper airway obstruction, bronchospasm, pneumonia, and exacerbation of chronic lung disease, though clinical documents records and telephone follow-up.

### DUS protocol

Diaphragm ultrasonograms were acquired on the right side pre-operatively and post-operatively with a Navi series ultrasonogram (Wisonic, Shenzhen, China) by an independent experienced anaesthesiologist who was blind to the TOFr results to avoid researcher bias. To ensure the reproducibility of the ultrasound examination, the location of the transducer was carefully marked, and the post-operative DUS examination was acquired at the same location within 2 minutes of extubation. The thickness of the diaphragm was assessed at the appositional zone of the diaphragm from images obtained at the 8-9th intercostal space on the anterior axillary line using a B-mode ultrasound with a 4–15 MHz sector array transducer at the end of inspiration and expiration. The DTF was calculated according to the following equation, during deep breathing (DB), pre-operatively (pre-DTF-DB) and post-operatively (DTF-DB).

$$\mathrm{DTF}=\frac{\mathrm{Thickness}\ \mathrm{at}\ \mathrm{the}\ \mathrm{end}\ \mathrm{of}\ \mathrm{inspiration}\hbox{-} \mathrm{Thickness}\ \mathrm{at}\ \mathrm{the}\ \mathrm{end}\ \mathrm{of}\ \mathrm{expiration}}{\mathrm{Thickness}\ \mathrm{at}\ \mathrm{the}\ \mathrm{end}\ \mathrm{of}\ \mathrm{expiration}}$$

The DE between inspiration and expiration was examined by M-mode ultrasonography with a 1–4 MHz curved array transducer from a subcostal area between the midclavicular and anterior axillary lines. The probe was directed cranially and dorsally, so that the ultrasound beam reached perpendicularly to the right diaphragmatic dome. Excursions during quiet breathing (DE-QB) and deep breathing (DE-DB) were assessed pre-operatively (pre-DE-QB and pre-DE-DB, respectively) and post-operatively (DE-QB and DE-DB, respectively). Two new parameters were defined, the diaphragm excursion fraction (DEF) and the diaphragm excursion difference (DED). These parameters were measured twice and averaged. The DEF was calculated as the DE-QB divided by the DE-DB, pre-operatively (pre-DEF) and post-operatively (DEF). The DED was defined as the DE-DB minus the DE-QB, and was also calculated pre-operatively (pre-DED) and post-operatively (DED).

### TOF monitoring

Acceleromyography (neuromuscular acceleromyography module; BeneVision N12, Mindray, China) was used to assess the acceleration of the adductor pollicis muscle after electric stimulation. After the skin was cleaned thoroughly, two surface electrodes were positioned over the ulnar nerve at the wrist of the dominant hand. The distance between the two electrodes was between 3 and 6 cm. An acceleration transducer was attached distally to the interphalangeal joint of the thumb. No preload was applied. The hand with the monitor was positioned on the bracket and securely fixed to prevent any movement of the fingers other than the thumb during each assessment. The skin temperature over the adductor pollicis muscle was maintained at > 32 °C. Following anaesthesia induction, the maximal response was obtained using single-twitch stimulation (2 Hz for 0.2-ms square wave) by gradually increasing the electrical current from 10 mA. A supramaximal response was triggered by an electrical current 20% above that which was necessary for a maximal response to reduce post-recovery drift. TOF patterns (a set of four supramaximal stimuli at 2 Hz for 0.2 ms) at 12-s intervals were applied to test the stability of baseline responses (variation of the TOFr < 5%) for 3 min. If baseline responses were unstable, the device was recalibrated [21]. The TOFr (T4:T1) was used to evaluate neuromuscular recovery. The TOFr within 1 minute before extubation was recorded. To prevent any bias, anaesthesiologists and ultrasound operators were blind to the TOFr results. PORC was defined as a TOFr at extubation of under 0.9.

### Statistical analysis

Sample size calculation was based on a preliminary experiment with a predictive sensitivity of 0.733 and predictive specificity of 0.667. With an alpha error of 0.05, beta error of 0.1, and no consideration of loss to follow-up, 75 patients were needed in current diagnostic test.

Patients with a TOFr value at extubation of over 0.9 comprised the non-PORC group, and the remaining patients comprised the PORC group (i.e. TOFr < 0.9). The discrimination performance of ultrasound parameters in identifying PORC was assessed using receiver operator characteristic (ROC) curve analyses, and the corresponding ROC curves were drawn using GraphPad Prism (GraphPad Software, Inc., State of California, USA). As DUS is rarely used peri-operatively to evaluate muscle function recovery, there are no well-accepted cut-off values of ultrasound parameters (DTF, DE, DEF, and DED) for the prediction of residual curarization. A higher area under the ROC curve (AUC) was considered as reflective of better test performance. The cut-off value was thereby identified by the point with the highest Youden index on the ROC curve to predict residual curarization, or equivalently, the highest sensitivity plus specificity. The sensitivity, specificity, positive likelihood ratio (LR+), negative likelihood ratio (LR-), positive predictive value (PPV), and negative predictive value (NPV), with corresponding 95% confidence intervals, were calculated at the cut-off value for each ultrasound parameter (DTF-DB, DE-DB, DEF, and DED). The Spearman correlation was used to evaluate the associations between the TOFr value at extubation and post-operative diaphragm parameters.

For all analyses, a two-sided *P*-value *<* 0.05 was considered significant. IBM SPSS Statistics 23.0 (Armonk, NY, USA) software was used for data analysis.

## Results

### Participants’ baseline demographic and clinical characteristics

**Fig. 1:**
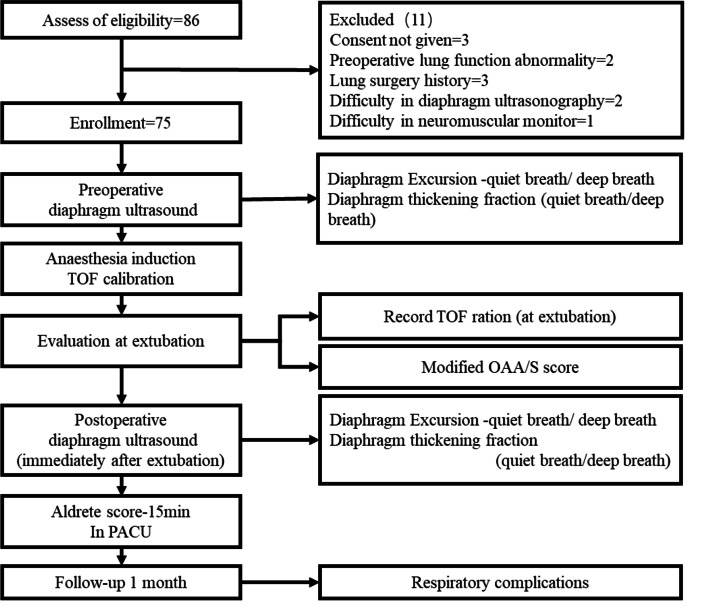
Recruitment and follow-up flow chart. Seventy-five patients receiving non-thoracic and non-abdominal elective surgery at the Peking Union Medical College Hospital from August to October 2019 were recruited; each provided signed informed consent before the diaphragm ultrasonogram. Baseline diaphragm ultrasound including diaphragm excursion and diaphragm thickening fraction of quiet breathing and deep breathing were acquired prior to operation. Abbreviation: PACU, post-anaesthesia care unit; TOF, Train of Four

A total of 86 patients (age, 39 ± 11 years) undergoing elective non-thoracoabdominal operations were invited to be assessed for initial eligibility between 1 August and 30 October, 2019. A total of 75 patients were finally enrolled in this study. Figure 1 shows the flowchart representing the patient enrolment process, the reason for excluding certain patients, and the procedure of the study. Fifty-two patients were assessed as ASA level I, and 23 patients as ASA level II. Residual curarization at extubation was identified in 41 patients (54.7%), according to the criterion of a TOFr at extubation of < 0.9. Patients were divided into PORC and non-PORC groups according to whether the TOFr was lower than 0.9 at extubation. Clinical data and DUS parameters are shown in Table 1. There were no significant differences in demographic characteristics, pre-operative diaphragm variables, fentanyl dose, or modified O/AAS scores between the two groups (Table 1).

**Table 1:** Clinical characteristics and baseline ultrasound indicators between patients with and without residual neuromuscular blockade

	non-PORC group (*N* = 34)	PORC group (*N* = 41)	*P*-value
Female (%)	58.8	73.1	0.131
Age^a^	36.4 ± 11.5	41.4 ± 10.2	0.065
ASA I (%)	73.5	65.9	0.616
Body mass index(kg/m^2)	22.95 ± 3.82	23.58 ± 3.01	0.429
Fentanyl dose^a^ (μg)	224.3 ± 93.8	212.8 ± 32.2	0.932
Rocuronium/weight^a^(mg/kg)	0.71 ± 0.15	0.74 ± 0.13	0.239
Anaesthesia time^a^(minute)	87.6 ± 30.9	73.2 ± 18.9	0.042
Pre-Thickening fraction^a^	0.50 ± 0.27	0.53 ± 0.23	0.252
Pre-Diaphragm excursion (QB)	1.53 ± 0.47	1.45 ± 0.43	0.466
Pre-Diaphragm excursion (DB)	4.88 ± 1.21	4.70 ± 1.14	0.518
Pre-Diaphragm excursion fraction	0.32 ± 0.10	0.32 ± 0.09	0.821
Pre-Diaphragm excursion difference	3.35 ± 1.11	3.25 ± 1.04	0.684
O/AAS-extubation^a^	1.2 ± 0.4	1.5 ± 0.5	0.155
TOFr at extubation	95.8 ± 7.5	53.4 ± 21.9	< 0.001
Thickening fraction^a^	0.44 ± 0.22	0.34 ± 0.18	0.039
Diaphragm excursion (QB)	1.48 ± 0.56	1.46 ± 0.52	0.868
Diaphragm excursion (DB)	4.11 ± 0.97	2.89 ± 1.37	< 0.001
Diaphragm excursion fraction	0.36 ± 0.12	0.56 ± 0.19	< 0.001
Diaphragm excursion difference	2.63 ± 0.82	1.43 ± 1.10	< 0.001

^a^ Abnormal distribution, Mann-Whitney rank sum test was used in group comparation

Abbreviations: *ASA* American Society of Anesthesiologists classification of physical status, *QB* quiet breathing, *DB* deep breathing

### Accuracy of DUS for the prediction of PORC

**Fig. 2:**
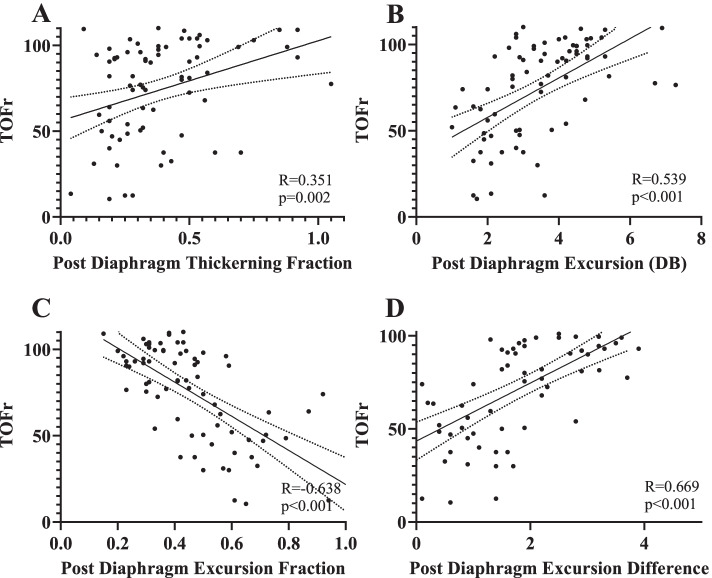
TOF ratio at extubation correlate with post-operative diaphragm ultrasound indicators. **A** Diaphragm thickening fraction (DTF), **B** Diaphragm excursion (DB), **C** Diaphragm excursion fraction (DEF), **D** Diaphragm excursion difference (DED). Abbreviation: DB, deep breathing; QB, quiet breathing; TOF, Train of Four

**Fig. 3:**
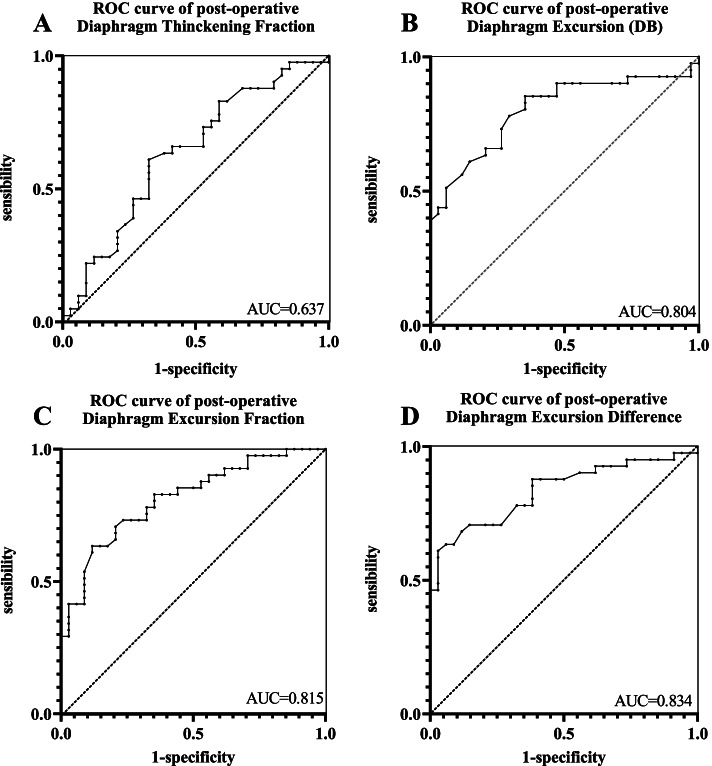
ROC curve of post-operative diaphragm ultrasound indicators. **A** Diaphragm thickening fraction (DTF), **B** Diaphragm excursion (DE), **C** Diaphragm excursion fraction (DEF), **D**. Diaphragm excursion difference (DED). Abbreviation: ROC, receiver operating characteristic curve; DB, deep breathing; QB, quiet breathing

DE-DB (*R* = 0.539, *P* < 0.001), and DED (*R* = 0.669, *P* < 0.001) were positively correlated with TOFr at extubation in moderate degree, while a weak correlation was found between DTF-DB and TOFr at extubation(*R* = 0.351, *P* = 0.045). DEF (*R* = -0.638, *P* < 0.001) was inversely correlated with TOFr at extubation (Fig. 2). Figure 3 shows the ROC curves of four DUS parameters for the prediction of PORC. Table 2 provides the cut-off values, sensitivity, specificity, LR+/−, PPV, and NPV of these DUS parameters. Among all these parameters, the DE-DB cut-off value for the prediction of the PROC was 3.88 cm, with a highest sensitivity of 85.4% (70.1–93.9%), specificity of 64.7% (46.4–79.7%), while, the DED cut-off value was 1.5 cm, with a highest specificity of 94.2% (80.3–99.3%), sensitivity of 63.4% (46.9–77.9%), LR+ of 10.78 (2.8–42.2), and PPV of 92.9 (76.9–98.1).

**Table 2:** Diagnostic accuracy of different diaphragm indicators after extubation including diaphragm thickening fraction (DB), diaphragm excursion (DB), diaphragm excursion fraction, and diaphragm excursion difference

	AUC (95% CI)	Youden Index	Cut-off Value	Sensitivity (%) (95% CI)	Specificity (%) (95% CI)	LR+ (95% CI)	LR- (95% CI)	PPV (95% CI)	NPV (95% CI)
DTF (DB)	0.637 (0.517–0.745)	0.286	0.33	61.0 (44.5–75.8)	67.7 (49.5–82.6)	1.88 (1.1–3.2)	0.58 (0.4–0.9)	69.4 (56.9–79.7)	59.0 (47.9–69.2)
DE (DB)	0.804 (0.703–0.905)	0.501	3.88	85.4 (70.1–93.9)	64.7 (46.4–79.7)	2.42 (1.5–3.9)	0.23 (0.1–0.5)	74.5 (64.5–82.4)	78.6 (62.7–88.9)
DEF	0.815 (0.708–0.895)	0.501	0.44	70.73 (54.5–83.9)	79.41 (62.1–91.3)	3.44 (1.7–6.8)	0.37 (0.2–0.6)	80.6 (67.5–89.2)	69.2 (57.6–78.9)
DED	0.836 (0.732–0.911)	0.575	1.5	63.4 (46.9–77.9)	94.12 (80.3–99.3)	10.78 (2.8–42.2)	0.39 (0.3–0.6)	92.9 (76.9–98.1)	68.1 (58.6–76.3)

Abbreviations: *AUC* area under curve of ROC curve, *CI* confidential interval, *LR+* positive likelihood ratio, *LR-* negative likelihood ratio, *PPV* positive predictive value, *NPV* negative predictive value, *DTF (DB)* Diaphragm thickening fraction (Deep breathing), *DE (DB)* Diaphragm excursion (Deep breathing), *DEF* diaphragm excursion fraction, *DED* Diaphragm excursion difference

### Clinical outcomes in the PORC and non-PORC groups

The modified Aldrete score was lower in the PORC group than in the non-PORC group (8.2 ± 1.2, 9.6 ± 0.7, *P* < 0.001), mainly because of a lower SpO_2_. There were no cases of airway obstruction, bronchospasm, pulmonary aspiration of gastric contents, apnoea, reintubation, unexpected ICU admission, atelectasis, or pneumonia in either group.

## Discussion

This is the first diagnostic test focusing on the use of DUS parameters to recognize PORC. Our findings suggest that DE-DB and DTF-DB are significantly correlated with the TOFr. Patients with residual curarization had a much lower DTF DB, DE-DB, DEF, and DED. In particular, the DED had a low sensitivity and high specificity in recognizing PORC.

### PORC and baseline DUS parameters

The PORC incidence in the current study was 54.6%, which is within the common range of published studies in China, but is higher than that in some recent American and European studies. Neuromuscular monitoring was performed by an independent investigator according to neuromuscular measurement guidelines. Both the anaesthesiologists and ultrasound operators were blind to the TOFr results to prevent researcher bias. DUS was performed immediately after extubation to shorten the time interval between the DUS and TOFr measurements. The DTF [12, 22] and DE [23] have been validated as repeatable and reproducible in recent studies. In addition, it is easier to detect the DE on right hemidiaphragm than on the left hemidiaphragm, and the result is more reproducible, according to previous research [24]. Thus, only right hemidiaphragm parameters were measured in this study.

Multiple factors (e.g. sex, age, BMI, and lung function) may influence DUS parameters [25]. However, no significant differences were found in sex, age, or BMI between the two groups in our study, which minimized selection bias. The baseline DE in our study was lower than that reported in French and Canadian studies [14, 24]. DE measurement was performed directly at the diaphragm dome to reduce the methodological weaknesses of our study. The difference in baseline DE may be due to the different ethnics and BMI of patient samples between our study(Asian, smaller BMI) and previous research (Most of Caucasian, higher BMI). Additionally, we found no significant differences between females and males, although many studies have shown sex differences in DE*.*

### Correlation between TOFr and diaphragm parameters

DUS is commonly used to assess diaphragmatic function recovery in the ICU [26, 27], but is rarely used in peri-operative situations. Only one clinical trial was designed to use both DUS and TOFr to evaluate the function of neostigmine and sugammadex as reversal drugs [26]. This study is the first to report correlations between DUS parameters and the TOFr. The TOFr was significantly and positively associated with DE-DB and DTF-DB in the bivariate correlation analysis (Fig. 2). This result does not contradict the traditional notion that the diaphragm is resistant to neuromuscular blockade, and diaphragm function clearly recovered earlier than did other muscular functions [28, 29]. We found no difference in DE-QB between the two groups, which suggests that early diaphragm recovery occurred in order to maintain the ability to breathe quietly. Additionally, we found that patients with PORC had a lower DE-DB and DTF-DB compared to patients who experienced full neuromuscular function recovery, indicating that diaphragm function was not fully recovered in the PORC group, and the ability to breathe deeply was compromised. Thus, the DE-DB and DTF can reflect compromised diaphragm recovery, as well as the curarization status. To rule out the influence of sedation [30], modified O/AAS scores were assessed, and no significant differences were found between the two groups. As a result, we defined the DEF and DED to represent the differences in diaphragm movements between QB and DB. Both DEF and DED were closely correlated with the TOFr.

The observed correlations provide insight into the diagnostic process of PORC. We compared the ROC curves of the DE-DB, DTF-DB, DEF, and DED. DED had the largest AUC (0.836, 95% CI: 0.732–0.911, *P* < 0.001), reported for the first time in the current study. The DED cut-off value was 1.5 cm, and had an relatively high specificity (94.1%) and low sensitivity (63.4%), as well as a high LR+ (10.78, 95% CI: 2.8–42.2) and high PPV (92.9, 95% CI: 76.9–98.1), when the PORC incidence was 54.6%, which means if a lower DED was detected,the possibility of PORC was 92.9%, but a normal DED cannot exclude PORC because of high false negative rate. These results can be attributed to the physiological characteristics of the diaphragm, since the diaphragm functionally recovers early after the administration of the neuromuscular blockade. DE-DB had a higher sensitivity (85.4%), thus the combination DE-DB and DED might provide better prediction of PORC, but need further study.

Although ultrasound parameters only achieved a best AUC of 0.836 and LR+ of 10.78 (for the DED), indicating barely acceptable accuracy, we still believe that the use of these parameters are highly clinical relevant. Ultrasound methods may serve as an important method for rapidly screening and monitoring for PORC, and help prevent complications due to PORC in surgical patients.

### Clinical perspective

Neuromuscular monitoring is still the gold standard for recognizing PORC, but it requires dedicated equipment, as well as precise calibration, and may incur discomfort in patients. Additionally, most Chinese centres only have a small number of neuromuscular monitor devices, insufficient for all patients under general anaesthesia, due to restricted medical funding. A DUS examination can help anaesthesiologists in detecting patients with PORC in the PACU and deciding whether or not to add an antagonist of neuromuscular functioning, without incurring discomfort in patients, when neuromuscular monitoring is not available. Due to the widespread use of ultrasound-guided regional blocks, one ultrasound device is usually available in most anaesthesiology departments in China. Therefore, this study can provide an alternative method for recognizing PORC, taking full advantage of the ultrasound devices available, rather than purchasing new neuromuscular monitor devices. In addition, DUS provides more insight into respiratory muscle function; thus, patients with respiratory disorders may benefit from DUS examination during the peri-operative period for early extubation.

### Limitations

Although a DUS examination has high reproducibility and feasibility, its quality and validity rely on the performance of the practitioners. Training and practice are required to master DUS skills [31]. In our study, DUS parameters were measured by one independent doctor who was blind to the TOFr result; thus, the influence of the operator was minimized, rendering the results more credible and homogeneous. However, this may restrict the generalizability of DUS application in recognizing PORC in the actual clinical conditions. Thoracic cardiac and abdominal surgery may affect diaphragm functions, which may influence post-operative DUS parameters. Additionally, the incision pain in these surgeries may restrict the voluntary movement of breathing [32, 33], and the incision site may affect ultrasound measurements; as a result, abdominal and thoracic surgeries were excluded from our research. Deep neuromuscular blockade is mostly required in abdominal and thoracic surgery [34]; as such, assessing how best to use DUS to identify PORC in patients undergoing abdominal and thoracic surgery warrants further research. Moreover, the muscle function recovery of the larynx is slower than that of the diaphragm, and this recovery is essential for maintaining an open upper airway [29]. As such, respiratory function integrity cannot be ensured, even if diaphragm function is fully recovered, due to the slow recovery of the laryngeal muscle. Finally, this study is an exploration with limited sample size, so further studies with larger sample size and variable clinical conditions are needed.

## Conclusions

Peri-operative DUS may be an additional method contributing to the recognition of PORC, with DED having high specificity.

## Data Availability

The datasets used and/or analysed during the current study are available from the corresponding author on reasonable request.

## Abbreviations

AUC: Area under the curve
ASA: American Society of Anesthesiologists
DB: Deep breathing
DED: Diaphragm excursion difference
DEF: Diaphragm excursion fraction
DTF: Diaphragm thickening fraction
DUS: Diaphragm ultrasound
DE: Diaphragmatic excursion
ICU: Intensive care unit
OAA/S: Observer’s assessment of alert/sedation
PUMCH: Peking Union Medical College Hospital
PACU: Post-anaesthesia care unit
post-: Post-operative
PORC: Post-operative residual curarization
pre-: Pre-operative
SpO_2_: Pulse oxygen saturation
QB: Quiet breathing
ROC: Receiver operator characteristic
TOFr: Train-of-four ratio
