# Correction to: Peri-operative diaphragm ultrasound as a new method of recognizing post-operative residual curarization

**DOI:** 10.1186/s12871-022-01565-0

**Published:** 2022-01-14

**Authors:** Jiaxin Lang, Yuchao Liu, Yuelun Zhang, Yuguang Huang, Jie Yi

**Affiliations:** 1 Department of Anesthesiology, Chinese Academy of Medical Science, Peking Union Medical College Hospital, No 1, Shuaifuyan, Dongcheng district, Beijing, 100730 China; 2 Medical Research Center, Chinese Academy of Medical Science, Peking Union Medical College Hospital, Beijing, 100730 China

**Correction to: BMC Anesthesiol 21, 287 (2021)**

**https://doi.org/10.1186/s12871-021-01506-3**

Following publication of the article [1], it was noted that in Table 1 postoperative diaphragmatic parameters (including thickening fraction, diaphragm excursion (QB), diaphragm excursion (DB), diaphragm excursion fraction, and diaphragm excursion difference) in PORC (Post-operative residual curarization) group and non-PORC group have been swapped with each other by mistake. Table 1 in this correction article is the correct version. We apologize for any inconvenience caused by this error.

**Table 1:** Clinical characteristics and baseline ultrasound indicators between patients with and without residual neuromuscular blockade.

	non-PORC group (*N*=34)	PORC group (*N*=41)	*P*-value
Female (%)	58.8	73.1	0.131
Age^a^	36.4±11.5	41.4±10.2	0.065
ASA I (%)	73.5	65.9	0.616
Body mass index (kg/m^2)	22.95±3.82	23.58±3.01	0.429
Fentanyl dose^a^ (μg)	224.3±93.8	212.8±32.2	0.932
Rocuronium/weight^a^ (mg/kg)	0.71±0.15	0.74±0.13	0.239
Anaesthesia time^a^ (minute)	87.6±30.9	73.2±18.9	0.042
Pre-Thickening fraction^a^	0.50±0.27	0.53±0.23	0.252
Pre-Diaphragm excursion (QB)	1.53±0.47	1.45±0.43	0.466
Pre-Diaphragm excursion (DB)	4.88±1.21	4.70±1.14	0.518
Pre-Diaphragm excursion fraction	0.32±0.10	0.32±0.09	0.821
Pre-Diaphragm excursion difference	3.35±1.11	3.25±1.04	0.684
O/AAS-extubation^a^	1.2±0.4	1.5±0.5	0.155
TOFr at extubation	95.8±7.5	53.4±21.9	<0.001
Thickening fraction^a^	0.44±0.22	0.34±0.18	0.039
Diaphragm excursion (QB)	1.48±0.56	1.46±0.52	0.868
Diaphragm excursion (DB)	4.11±0.97	2.89±1.37	<0.001
Diaphragm excursion fraction	0.36±0.12	0.56±0.19	<0.001
Diaphragm excursion difference	2.63±0.82	1.43±1.10	<0.001

^a^Abnormal distribution, Mann-Whitney rank sum test was used in group comparation

*Abbreviations*: *ASA* American Society of Anesthesiologists classification of physical status, *QB* quiet breathing, *DB* deep breathing.

The original article [1] has been updated.
